# Ambulance Arrangements for Hospitals

**Published:** 1910-11-12

**Authors:** 


### THE QUESTION BOX AT THE GLASGOW CONFERENCE.

[Discussions on Important Subjects at the recent Conference of the British Hospitals Association held at Glasgow.]

### I.—AMBULANCE ARRANGEMENTS FOR HOSPITALS.

Mr. Nicol Paton Brown: Mr. Chairman, ladies and gentlemen, I have been asked, as Chairman of Council of the St. Andrew's Ambulance Association, by way of opening the discussion on the question, "What are the best ambulance arrangements for hospitals, and is the motor-ambulance quite reliable?" to say a few words regarding the arrangements of the St. Andrew's Association and our experience of horse and motor waggons. I shall confine my remarks specifically to the economic question of the relative working costs of motor as compared with horse waggons. In our waggon service we own and employ five horse-waggons drawn by pairs of horses, three pairs of horses being kept exclusively for ambulance work, two pairs ready harnessed during the day and one pair during the night. The other two waggons can be horsed and brought out promptly in cases of a sudden run of accidents or in cases of a sudden disaster. One trained ambulance attendant goes out along with the driver of each waggon. We have found the horse-waggons most reliable and efficient, but with the advent of the motor-car in the streets or the highway it was thought, four years ago, that an experiment should be made with a motor-waggon to see if an improvement could not be made in our service by getting more quickly to the scene of an accident, and when there by carrying the patient more comfortably as well as expeditiously to the hospital. We accordingly purchased a motor-waggon, and as it was the first to be made in the country we had some difficulty in getting any maker to undertake the work, as one of our conditions was the placing upon the chassis of a replica of our standard horse-waggon, which had been found so serviceable in use and so well planned. The great difficulty was to get the chassis adapted for the body of the waggon, and perhaps we made a mistake in not rather making the body to fit a standard type of chassis. The result was that we got an abnormally long and heavy motor-ambulance, which has proved costly to run, rather difficult to handle in the streets, and consequently not very suitable for accidents in town. Still the experience gained has been valuable, and whilst we are satisfied that so far horse-waggons are cheaper to run and more reliable, we are now about to replace our motor by a new one, lighter in weight and thoroughly up to date in type of engine and chassis, and with a body designed to fit the standard type of chassis; and on this account we have reduced the length of the ambulance waggon, and hope thereby to be able to handle it more easily and quietly in traffic. The cost of the horse-waggons works out at 1s. 0½d. per mile, all charges included except depreciation, against 1s. 5d. per mile for the motor-waggon. The cost of the motor-waggon is thus 4½d. per mile more than the horse-waggons; and, if depreciation were counted in each case, the difference in cost would be still more in favour of the horse-waggon. The motor-waggon has been greatly in demand by doctors, especially country doctors to send sick patients in to the town hospitals; the getting into and out of trains and the bustle at the stations is thus altogether avoided, the patient being conveyed quietly from door to door. Finally, as to reliability, our experience is that our motor has not been quite reliable. It has been subject to many mechanical breakdowns, having been off the road during the three years of our running from sixty to seventy days on the average during the year. Speaking from personal experience as a motor-car owner, I am confident that with our new ambulance motor we shall get very much better results as regards reliability and also, I may say, as to cost. Sir George Beatson, from his professional experience as a hospital surgeon, as well as the head of the Council of the Ambulance Association, will be able to give you a much more comprehensive view of the subject-matter of the question asked than I can pretend to do, from my lack of medical and surgical knowledge as well as my shorter experience of the work of the Association.

The following is the motor-ambulance record of work done in three years and nine months:—

| — | One Year to Sept. 30, 1907 | One Year 1908 | One Year 1909 | Nine Months 1910 |
|---|---|---|---|---|
| Miles run ... ... ... | 6,433 | 3,367 | 4,719 | 4,167<br>1 yr. 5,856 |
| Patients carried—accidents . | 172 | 35 | 28 | — |
| Sick removals ... ... ... | 342 | 118 | 156 | — |
| Days out ... ... ... | 227 | 125 | 132 | — |
| Days in for breakdowns and repairs ... ... ... | 56 | 28 | 79 | — |
| Maintenance; total cost excluding depreciation ... | £422 | £274 | £311 | — |
| Per mile ... ... ... | 1s. 3¾d. | 1s. 7½d. | 1s. 3¾d. | — |
| Miles per gallon petrol ... | 6.50 | 5.95 | 7.06 | |
| Running costs, petrol, oil, and tyres ... ... ... | — | £149 | £169 | — |
| Per mile ... ... ... | — | 10.67 | 8.63 | — |

Costs of horse-ambulances, years 1906 to 1907, average per waggon:—

| | |
|---|---|
| Miles run ... ... ... ... ... | 3,982 |
| Accidents ... ... ... ... ... | 521 |
| Removals ... ... ... ... ... | 376 |
| Cost of maintenance, excluding depreciation ... ... ... ... ... | £204 |
| Per mile ... ... ... ... ... | 1s. 0½d. |
| Earnings ... ... ... ... ... | £78 |

Five horse-waggons are kept, three pairs of horses reserved exclusively for ambulances; two pairs kept ready harnessed during day, one pair kept ready during night.

Sir George Beatson: Mr. Chairman, ladies and gentlemen, I am glad this subject has come before the Conference, because I think it is one that is of great importance both to hospitals and the community generally. I think this fact is self-evident when you remember that a city ambulance service has to deal, first of all, with accidents as they occur in the streets or as they occur in our public works or even in private dwellings. Secondly, it has to deal with the removal of the sick—it may be to nursing homes or it may be to hospitals—and also under the sick I would include the infirm, who have to be taken from one house to another at a time when they have to change their residence, or, as we call it in Scotland, "flitting." To the hospitals this matter is essentially of importance, because it makes the greatest difference to them whether their patients arrive there promptly after an accident and in a satisfactory state. These are two factors which materially affect the length of the residence of the patient in the hospital, and consequently the result. The same holds good with the community. If any of us should unfortunately receive an accident on the street or anywhere, we want to be properly dealt with, and also to be taken as comfortably as possible to the nearest and best medical assistance, which can always be got at the infirmary. An infirmary in Scotland means the hospital. This leads me to say that any city ambulance service to be efficient must possess four features: first of all, it must be prompt in response to calls; secondly, it must furnish rapid and comfortable transport; thirdly, it must bring, if possible, with it a proper attendant trained in his duty to attend the sick and injured; and, fourthly, it must be economical. These are four points that I consider are essential in any city ambulance service. We are all agreed upon this; but before establishing such a service we come to this very important preliminary point: Is such service in the city to be carried on officially and rate-supported, or is it to be a voluntary one—the outcome of the good-heartedness and the liberality of the public? That is a very important point that has to be settled. So important is it, that in London they have been about two years trying to settle it, and have not yet arrived at a conclusion. We in Glasgow have not taken so long about it, because for twenty-eight years we have established a system founded on the voluntary system, which has been very successful, and my view is in favour of having a voluntary system of ambulance service in any large town. Let us take, then, the four points that I have mentioned. First of all, there is the promptness in response to a call. That has been now completely revolutionised by means of the telephone, and with the aid of the telephone there can be given a rapidity of response that I think should meet all demands. Our experience has been that most accidents happen between 6 A.M. and 6 P.M. in the streets, and there is not a shop or house where you have not a telephone immediately at your call. The lesser number of accidents are from 6 P.M. to 6 A.M., and these are very few in the streets and chiefly occur in works where a nightshift is going on. These works have all their telephones, so that there is no delay. It is on the telephone that our service must rely to secure promptness. I can remember a case where our ambulance-waggons were on the scene almost before the accident happened. It occurred with a runaway tramway-car in Renfield Street, and a man saw what was going to happen, and telephoned to the ambulance office to say that there was going to be a very severe accident. Our ambulances are housed close to the spot, and they were there practically at the time the accident happened. As to the rapid transport, this raises a question whether we should rely on motors or on horse-ambulance waggons, and I confess myself that, from the experience we have had over a good many years, and from what I have learned from Colonel Burns, while the motor is more rapid, you have not the reliability that you have with the horse-ambulance, and, taking it all round, I think you must rely upon the horse-ambulance in the present state of motor construction. Then, as regards comfort, the horse-ambulance may still require some improvement, but our Glasgow waggon of low construction and with all its various springs has been the result of a great deal of thought, and it is better than any other waggon has shown itself in the past that I know of. I think the motor-ambulance is very comfortable as regards vibration and so on, but the horse-ambulance is not behind. Then, thirdly, as regards trained assistants at the time of accident, I think that is entirely met by having a trained attendant who has passed through a proper course of instruction and has had experience and is watchful and thoughtful. I think our Secretary has had no complaints of anything like inefficiency on that point. You must also remember that, as regards street accidents, all our police in Glasgow have gone through a course of first aid, and their services are very valuable to us. As regards economy, you must include all the sums in connection with the cost of the ambulance turnout. If you take the whole expense of our rent and salaries, and all that sort of thing, it amounts to a good deal; and, if I remember rightly, in previous years Colonel Burns worked it out at 8s. 2d. for each turnout, and we have an average of about ten or twelve turnouts per day. We have perhaps 2,000 turnouts in a year, and, till I am satisfied that the motor will do more than it has done in the past, I think we ought to continue as we are doing.

Dr. Goodall: Mr. Chairman, ladies and gentlemen, I am sorry that I had not time before leaving London to look up some points of detail that would have been interesting to this meeting; but I should like to say that in London the only ambulance service we have is a motor service for the removal of infectious cases, and this service has been running for the best part of thirty years. I think I can claim that we have an exceedingly well-organised service. I am surprised to hear Sir George Beatson say that you have not been satisfied with the motors. I was making inquiries over the telephone in our ambulance department on the very morning I came up here, so as to be certain on some points, and I was informed that our board have decided to do away with horse-ambulances altogether and have entirely motor-ambulances. They have been trying different motors for some years, and they have come to the conclusion that the petrol is the best. We have as many as twenty ambulances out at once just now—and they are all motor-ambulances. There are six ambulance stations attached to the large fever hospitals in London, and I am told by the chief clerk that by adopting the motors they will be able to do away with two of these, thereby reducing the cost of the upkeep, and there is to be a considerable reduction in the staff on that account. Then we have motors for removing convalescent patients for about ten miles, and we can get the work done in about half the time we could before. I understand, from the remarks I have heard to-day, that the motor-ambulance sometimes breaks down, and that is unsatisfactory. I would unhesitatingly say that our delays caused by breakdowns of the motors have not been any more frequent than with the horse-ambulances. If anything goes wrong in London the driver has instructions to telephone to the nearest station to send another motor; but, so far, there have been very few accidents to the motors.

Dr. Robertson (Leith): Mr. Chairman, ladies and gentlemen, I will be particularly brief. I am sorry to hear from Sir George Beatson about the motor-ambulance, and I am afraid that his type of motor must have been an obsolete one. Councillor M'Pherson can bear me out when I say that the efficiency of the motor-ambulance which we use is supreme compared with that of the horse-ambulance. My experience is that after our motor-ambulances have been running for thousands of miles the number of breakdowns was infinitesimal as compared with what they had been with the horse-ambulance. The petrol engine has come to such a state of perfection that it is absolutely reliable. In regard to economy nowadays, we can secure removable bodies which can be placed on the chassis. We have several bodies suspended from the roof of the house, so that they can be put down for different diseases. I am glad that this subject has been ventilated, if it were for nothing else than to indicate that some of us have not lost faith in the petrol waggon, which has proved itself quite reliable.

Councillor John M'Pherson (Edinburgh): Mr. Chairman, ladies and gentlemen, five years ago Edinburgh got its first motor-ambulance, and it was very unsatisfactory for a long time, as the breakdowns and the repairs were frequent. The second ambulance we got gave absolute satisfaction, and we have recently got a third.

Mr. Nicol Paton Brown: Mr. Chairman, ladies and gentlemen, I only wish to say in reply that we have by no means lost faith in the possibilities of motor-ambulances.

